# Prefrontal-posterior coupling mediates transitions between emotional states and influences executive functioning

**DOI:** 10.1038/s41598-019-44624-2

**Published:** 2019-06-04

**Authors:** Yu Hao, Lin Yao, Derek M. Smith, Edward Sorel, Adam K. Anderson, Eric H. Schumacher, Gary W. Evans

**Affiliations:** 1 Department of Design and Environmental Analysis, Cornell University, Ithaca, NY USA; 2 School of Electrical and Computer Engineering, Cornell University, Ithaca, NY USA; 3 School of Psychology, Georgia Institute of Technology, Atlanta, GA USA; 4 Psychology Resource Group, Ithaca, NY USA; 5 Department of Human Development, Cornell University, Ithaca, NY USA

**Keywords:** Stress and resilience, Prefrontal cortex, Cognitive control

## Abstract

Emotions often result from fluctuating experiences with self-regulation unfolding over time. However, most research has been focused on neural responses to static, affective stimuli. We studied emotion transitions, which correspond to dynamic conditions of varying affective valence or intensities. Functional coupling of prefrontal and posterior cortex (EEG coherence) was recorded during exposure to stable versus changing emotion-eliciting images (static vs. dynamic conditions). Prefrontal-posterior coupling was decreased in the dynamic conditions compared to the static conditions. A decrease in prefrontal-posterior coupling implies less control of the prefrontal cortex over perceptual information, which may allow the brain to become more affected by emotional fluctuations. We also assessed the aftereffect of EEG coherence on executive functioning, utilizing the flanker task. Among individuals reporting higher chronic stress, executive functioning decreased after dynamic conditions. This decrease in executive functioning was mediated by the decrease in prefrontal-posterior coupling in the dynamic conditions. These findings suggest that the strength of prefrontal-posterior coupling is not only related to emotional transitions but also to executive functioning. The deterioration of executive functioning after dynamic emotional processing may reflect the additional cognitive effort required to process dynamic shifts in affective stimuli, and this relationship is exacerbated by chronic stress.

## Introduction

Throughout their daily lives, humans continuously interact with a dynamic environment. These dynamic interactions require constant updating of internal representations and respective behavioral adjustments. One important aspect of this constant updating is changes in emotional states. These emotion dynamics not only reflect momentary and transient responses to current stimuli but are also influenced by context and individual differences in self-regulation, which are crucial for psychological health^1–3^.

However, to date, most emotion research has focused on responses to affective stimuli from a static perspective. Little is known about the brain’s responses to changing emotional experiences nor how such responses are related to self-regulation mechanisms. Our study investigates hypothesized differences in the brain’s responses to static vs. dynamic emotional conditions and their influence on performance in a subsequent executive functioning (EF) task. Because changing stimulation conditions can trigger emotion dynamics^4–7^, in the present study, we define emotion transitions as responses to dynamic stimulation conditions with varying affective valence or intensity, e.g., image sequences with changing valence and/or intensity, as opposed to static conditions without affect transition.

The modulation of emotional responses inherently entails processes of self-regulation. Self-regulation involves a volitional component mediated through EF (working memory operations, behavioral inhibition, and task-switching) as well as a nonvolitional component enabled by autonomous systems^8–10^. During dynamic changes in the emotional content of our environment, the residual affective processing from previously experienced emotional stimuli likely affects the perception of current emotional stimuli. Therefore, when emotion transitions occur, it is reasonable to hypothesize that greater EF processing is recruited in the prefrontal cortex (PFC) in order to deal with potentially conflicting responses due to an overlap in incompatible emotions^11^. If this is the case, greater demands upon EF would be expected when individuals are exposed to dynamic as opposed to static emotional stimuli.

Importantly, self-regulation in response to emotional stimuli may be impaired by a large range of factors including, amongst others, chronic stress (e.g.^9^). Chronic stress has well documented effects on behavioral indices of EF^12,13^. Moreover, at the level of the brain, chronic stress is also known to cause structural and functional changes in areas underlying EF like the PFC and the hippocampus^12,14–17^. These chronic stress-induced changes in behavior and brain likely interfere with regulatory functions that facilitate responding to acute demands. As a result, chronically stressed individuals may exhibit more reactive rather than reflective psychophysiological responses, which in turn, place even greater demands on self-regulation^8^. Thus, it may be assumed that dynamic emotional stimuli may result in cumulative aftereffects, which might be different from the effects of purely static negative events on EF. Therefore, investigating the role of chronic stress on EF after emotion transitions may provide important insights on individuals’ capability to recruit EF during emotion transitions.

We tested these ideas using a well-established EEG functional connectivity measure: cross-regional cortical synchronization. This measure indexes functional communication among brain areas and is believed to reflect top-down executive control over incoming perceptual information. Specifically, EEG coherence between prefrontal and posterior cortical regions increases during exposure to emotionally arousing or threatening stimuli, presumably protecting the individual from aversive input^18^. A reduction in EEG coherence between prefrontal and temporoparietal regions may reflect a relative loosening of executive control over the more posterior, predominantly perceptual areas. Research on individual differences in brain responses to affectively laden information has shown that decreased prefrontal-posterior coupling during social-emotional stimulation is related to greater influence of perceptual input on affective states^19–27^. For example, individuals with high trait rumination exhibited decreased prefrontal-posterior coupling during negative emotional stimulation and the persistance of negative affect was related to the degree of prefrontal-posterior decoupling^26^. Similarly, prefrontal-posterior decoupling is related to the development of intrusive memories in response to viewing negative emotional stimuli^25^. These empirical findings indicate that the investigation of prefrontal-posterior coupling during affective stimulation may provide critical insights on how control and self-regulatory mechanisms undergird emotional processing. As dynamic emotion transitions likely involve the processing of previous stimuli, extending the Papousek and colleagues’ findings might be particularly illuminating for understanding self-regulation during dynamic emotional stimulation.

In the present study, participants watched image sequences randomly presented with different patterns (conditions), some of which were static image sequences (all neutral or all negative images), and others which were dynamic image sequences (transition from neutral to negative or vice versa). Immediately after each image sequence, EF was assessed with a flanker task. EEG coherence was recorded during both emotional stimuli and the flanker task. Considering the EEG coherence findings outlined above^18–27^, we calculated coherence in the beta frequency band ([14 30] Hz). We also specifically investigated beta 1 range (14 20 Hz), as it has been implicated in higher-level cortical processing (e.g.^28–30^). We hypothesized that (1), decreased prefrontal-posterior coherence would occur in dynamic emotion transition conditions compared to static emotional conditions. This hypothesis is based on the assumption that dynamic stimuli elicit higher self-regulation demands which consume executive resources, making individuals more susceptible to perceptual input. To corroborate this assumption, we also hypothesized that (2), EEG coherence would mediate the relationship between emotional stimulation and subsequent EF performance. More precisely, we expected that especially in dynamic conditions, decreased prefrontal-posterior coupling would impair performance on the subsequent flanker task and thus reflect the aftereffects of EF resource depletion. (3) This link between EEG coherence, emotional stimulation and EF should be even stronger among individuals experiencing elevated chronic stress, given that their cognitive resources are strained from the beginning.

## Results

### Coherence in static vs. dynamic conditions

**Figure 1:**
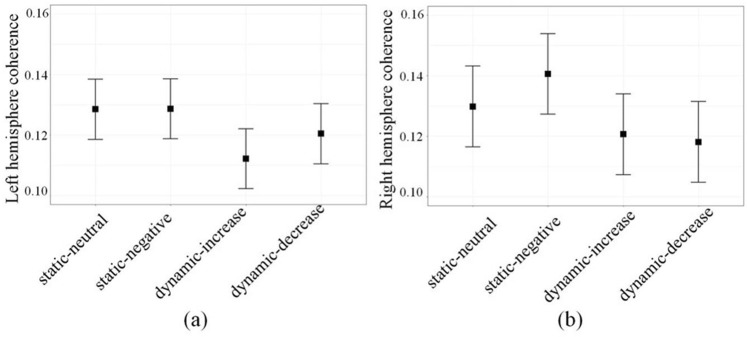
Beta 1 frequency range prefrontal-posterior coherence of four image sequence conditions in left (**a**) and right (**b**) hemispheres. Error bars are standard errors.

We first examined the effect of static and dynamic image sequence conditions on EEG prefrontal-posterior coherence. Image sequence conditions significantly affected coherence in beta 1 range [14 20] Hz and beta range [14 30] Hz (p < 0.001). Yet, only Beta 1 coherence mediated the relationship between the image sequence conditions and subsequent measures of EF. For this reason, our report will focus on beta 1 coherence only. As shown in Fig. 1(a,b), significant effects of image sequence condition on beta 1 coherence were observed in both hemispheres (right hemisphere, *F (3, 96)* = 13.00, *p* < 0.001, proportion change of variance (*PCV*) = 26.7%; left hemisphere, *F (3, 96)* = 7.22, *p* < 0.001, *PCV* = 15.9%). Planned comparisons indicate that beta 1 coherence was higher during the threatening static-negative than during the static-neutral stimulus in the right hemisphere (pairwise comparison: Estimate = 0.0146, *S.E*. = 0.0066, *p* < 0.05).

Combining the static conditions together and comparing them to the combined dynamic conditions, significantly lower coherence in the dynamic vs the static conditions was observed in both hemispheres (right hemisphere, *F (1, 98)* = 28.49, *p* < 0.001, *PCV* = 21.7%; left hemisphere, *F (1, 98)* = 18.26, p < 0.001, *PCV* = 14.9%). Table 1 shows statistics of beta 1 coherence.

**Table 1:** Estimates (standard errors) of static condition vs. dynamic condition.

	Condition		
	Dynamic	Static	Dynamic-Static
Right hemisphere 95% CI	0.12 (0.01) [0.10, 0.14]	0.14 (0.01) [0.12, 0.16]	−0.02 (0.00)
Left hemisphere 95% CI	0.12 (0.01) [0.10, 0.14]	0.13 (0.01) [0.11, 0.15]	−0.01 (0.00)

There were no main or interactive effects of chronic stress (*M* = 15.91, *SD* = 6.33) on coherence. Chronic stress was also unrelated to flanker performance (*F* (24) = 0.73, *p* = 0.40) or frontal-posterior coherence (*F* (31) = 3.00, *p* = 0.09).

### Coherence as a mediator of condition effects on flanker

**Figure 2:**
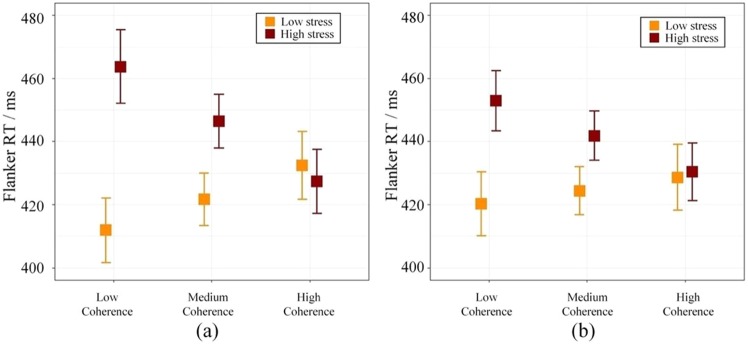
Flanker RTs (congruency effect) by prefrontal-posterior beta 1 coherence during image sequence (**a**) and flanker task (**b**) in the right hemisphere.

We next evaluated the mediating role of coherence on the relationship between emotional stimuli and executive functioning. Before testing mediation, we examined how coherence influenced flanker performance (congruency effect). Coherence interacted with chronic stress on subsequent flanker performance in both hemispheres (right, *F (1, 53)* = 17.70, *p* < 0.001, *PCV* = 15.6%; left, *F (1, 64)* = 10.99, *p* = 0.002, *PCV* = 7.0%). Under high chronic stress, decreased coherence during image sequences led to worse flanker performance, whereas increased coherence facilitated flanker performance (see Fig. 2(a)).

Coherence during image sequence was highly correlated to coherence during the flanker task (*r* = 0.90, *t* (130) = 24.16, *p* < 0.001). Furthermore, coherence during the flanker task interacted with chronic stress on flanker performance in the right hemisphere (*F* (1, 68) = 4.89, *p* = 0.030, *PCV* = 16.9%). Left hemisphere coherence was not significantly affected (*F* (1, 79) = 0.24, *p* = 0.63). Under high chronic stress, lower coherence during the flanker task led to worse flanker performance, whereas higher coherence facilitated flanker performance (see Fig. 2(b)).

**Figure 3:**
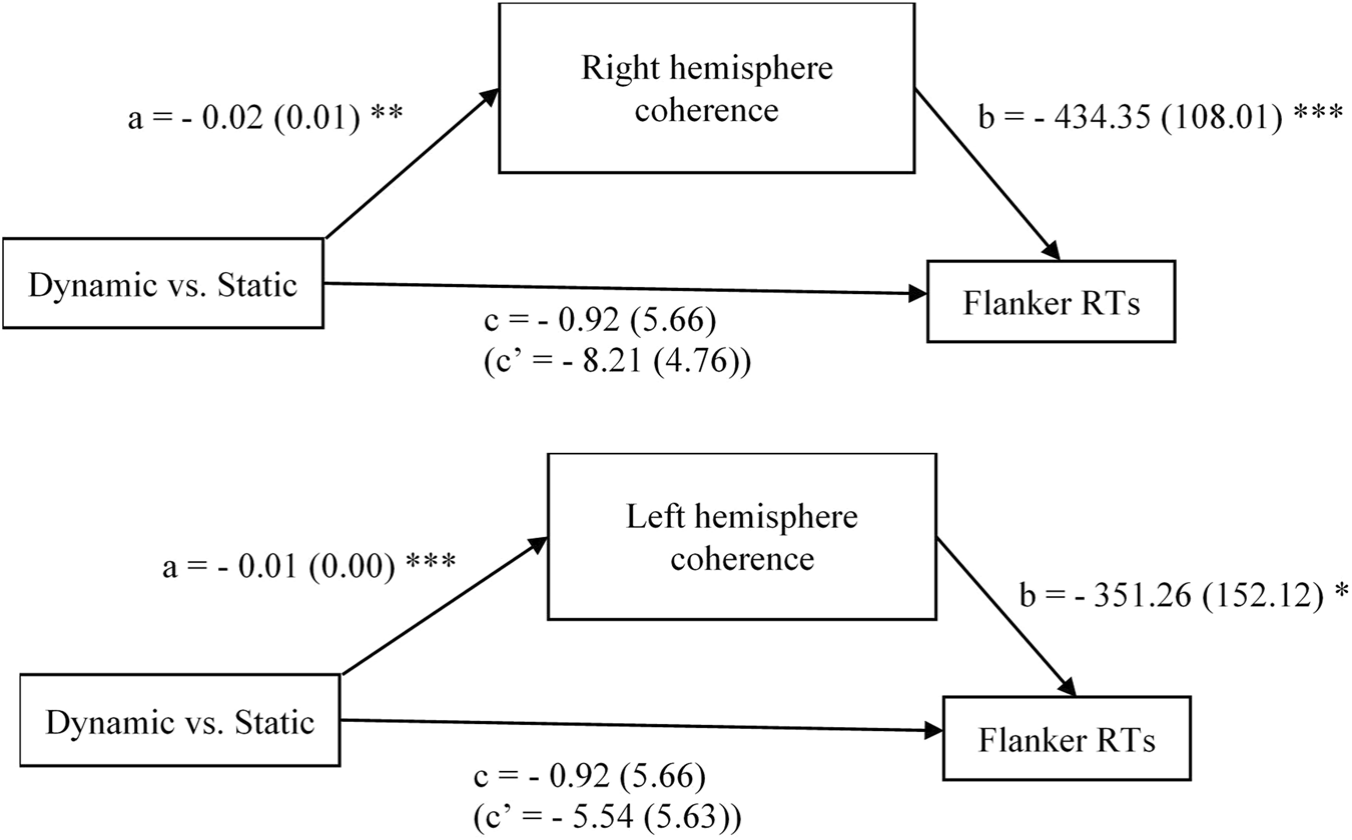
Mediation results—beta 1 frequency range prefrontal-posterior EEG coherence mediates the effects of emotion stimulation conditions on flanker RTs. Estimates (standard errors), *p < 0.05, **p < 0.01, ***p < 0.001.

The effect of static vs. dynamic emotional conditions on flanker performance was mediated by beta 1 coherence, but only in participants reporting high chronic stress (above the mean) (Fig. 3). In the right hemisphere, the indirect path was *ab* = 7.91, with a standard error of 3.20 (*p* = 0.013); in the left hemisphere, the indirect path was *ab* = 5.01, with a standard error of 2.48 (*p* = 0.044). In participants high in chronic stress, the dynamic conditions evoked lower coherence, which led to slower reaction times (RTs).

## Discussion

The present study investigated prefrontal-posterior coupling during exposure to both static and dynamic affective image sequences and its link to executive functioning. Decreased coupling between prefrontal and posterior cortical regions was observed during emotion transitions regardless of affect (neutral or negative) and also impaired subsequent executive functioning. However, this effect only occurred among individuals high in chronic stress. Furthermore, the apparent adverse impact of processing dynamic emotion stimuli on EF was mediated by EEG coherence in beta 1 range ([14–20] Hz) during image viewing.

Our data replicate prior research showing that negative stimuli can elevate prefrontal-posterior beta coherence^18^, but this is the first demonstration that beta ([14–30] Hz), including beta 1 range ([14–20] Hz) coherence is related to emotion transitions. Decreases in prefrontal-posterior EEG beta coherence induced by emotional stimulation reflect a decrease in the prefrontal cortex’s regulatory control over parietal regions^21–26^. Therefore, beta coherence plays a critical role in brain responses to emotion-cognition interaction.

In the present study, we interpret the changes in prefrontal-posterior coupling during dynamic sequences as a neural correlate for self-regulation. In line with this interpretation, our results showed that EF was hindered immediately after exposure to dynamic emotional image sequences. While only constituting indirect evidence, this finding suggests that emotion transitions elicit increased self-regulation demands, which occur at the expense of other executive functioning, the aftereffects of which may be visible in impaired performance in the subsequent flanker task. Importantly however, this effect was only found in participants reporting high chronic stress. Accordingly, it appears that people with chronic stress are more vulnerable in contexts demanding self-regulation. One possible mechanism for this relationship is that residual emotion from preceding images might generate emotional conflicts, mobilizing more executive control mechanisms for self-regulation in order to manage the conflicting emotional stimuli. It also makes sense that chronically stressed individuals might be less able to manage conflicts^31^, given already impaired attentional control^17^. This also appears to diminish at least short term EF capacity as indexed by slower reaction times in the flanker task in the present study^9,32^.

Importantly, our study calls attention to the mediating role of brain synchronization in the relationship between emotion transitions and EF. The reduced coherence evoked by emotion transitions continued during the flanker task, which suggests that individuals reporting high chronic stress were vulnerable to high demands for attentional allocation during emotional image viewing. This is consistent with prior work showing that chronic stress disrupts functional connectivity within the frontoparietal network that mediates attention shifts^17^. Another possible explanation is that the reactive rather than reflective disposition of those with high chronic stress levels may have weakened prefrontal cortical regulation of amygdala responses^33^. This might sustain experiences of emotion in the absence of an emotional stimulus. Note that the significant mediation effect was found only with beta 1 coherence but not the entire beta range, which might be age-related^34^, as our participants were all college students.

The primary limitations of the current study are that the duration of the induced drop in EF performance is unknown because only a limited number (6) of flanker trials were used to test for aftereffects. It is also unknown whether other negative emotional sequences would create similar results. A valuable extension of this study would be to examine individuals with self-regulatory difficulties, for instance, individuals with anxiety disorders. Several strands of evidence show that PFC dysfunction reported in mental disorders such as depression or anxiety, manifests in diminished attentional/inhibitory control during cognitive tasks and sustained negative affect processing^35–38^. Moreover, evidence of EEG prefrontal-posterior coherence reduction during emotional perception also comes from studies on individuals with schizotype^22^ and individuals with gelotophobia with/without schizophrenia spectrum/social phobia^21^. Understanding brain response to emotion transitions may inform our understanding of the cognitive capacity required to regulate and control emotional episodes. Hence, studies manipulating self-regulation capability by EF training^9,39^ or cognitive reappraisal^40^ under emotion transition events are needed.

More speculatively, the changes in brain activity uncovered in the present study might explain why exposure to fluctuating emotional context, be it positive or negative, correlates with poorer psychological health^41^. In this respect, it may be helpful to extent investigations of neural substrates underlying dynamic emotion transitions to other dynamics, such as other valence changes, or multiple transitions within one affect stimulation. In addition, the right and left hemisphere exhibited different coherence pattern across four conditions (Fig. 1). Additional work should investigate neural response changes during dynamic conditions in each hemisphere. Such endeavors could inspire more complex models of the neurodynamic of emotions and may also facilitate insights on neurocognitive markers of psychiatric disorders^42^.

## Methods

### Participants

Forty right-handed students from Cornell University were recruited. Informed consent was obtained, and participation was compensated with course credits or $20 cash. Due to incomplete data, 33 participants were included in the final sample (51.5% females, age *M* = 22.40, *SD* = 3.80). Exclusion criteria were any open or healing wounds on the scalp, use of any medication that could affect the nervous system and any history of neurological disorders. Participants were requested to refrain from alcohol caffeine, and other stimulants for four hours before the experiment. They were also asked to sleep for at least six hours the night before the experiment. The study was approved by Cornell’s Institutional Review Board and was performed in accordance with its guidelines. Informed consent was obtained from all participants.

### Procedure

To measure chronic stress, we administered the Perceived Stress Scale (PSS) before EEG recordings^43^. PSS was designed to measure perceived stress level, i.e., unpredictable, uncontrollable, and overloaded. This questionnaire contains 10 items and requires respondents to rate the degree to which situations are appraised as stressful in the last month, such as: “In the last month, how often have you felt confident about your ability to handle your personal problems?” or “In the last month, how often have you felt difficulties were piling up so high that you could not overcome them?”. PSS is a reliable and valid instrument for the assessment of perceived stress in college students and workers^44,45^.

**Figure 4:**
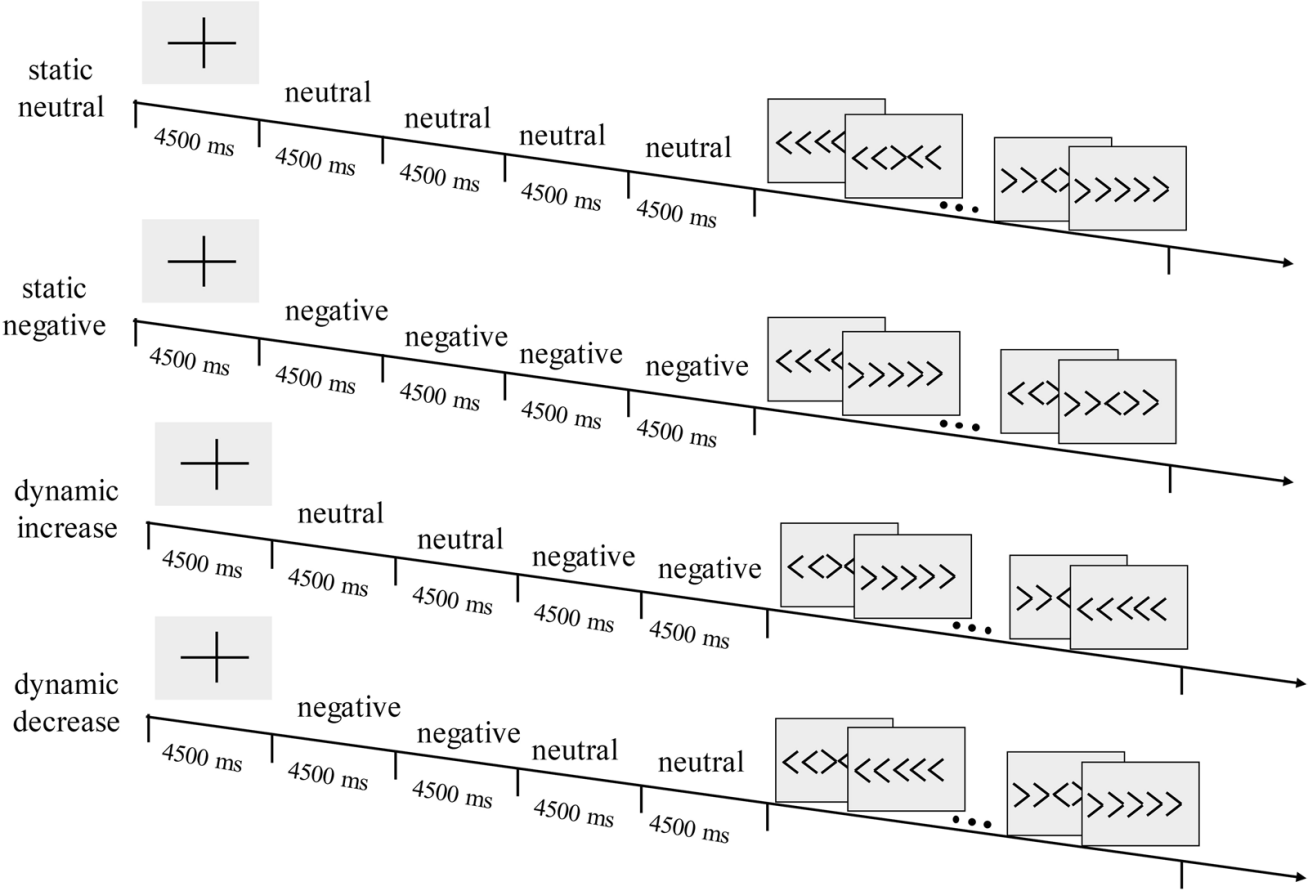
Schematic representation of the four image sequence conditions directly followed by the flanker task.

Affective state was manipulated by 96 negative, emotionally threatening images (valence: *M* = 2.37, *SD* = 0.65, arousal: *M* = 5.95, *SD* = 0.77) and 96 low arousal neutral images (valence: *M* = 5.12, *SD* = 0.53, arousal: *M* = 3.17, *SD* = 0.66) selected from the IAPS database^46^. Four images, each lasting 4500 ms, were presented one by one as an image sequence. As Fig. 4 shows, four different types of image sequences were defined as experimental conditions. For static conditions, the sequences consisted of all neutral (static-neutral) or all negative (static-negative) images, which appeared 6 times each. For dynamic conditions, the first two images and the last two images were from the same affect category, either neutral or negative; therefore, the transition occurred at the third image. The images either transitioned from neutral to negative (dynamic-increase) or from negative to neutral (dynamic-decrease), which appeared 18 times each. The *image sequences* of all conditions were randomly presented with no image repetition to participants. Due to the randomization of dynamic and static conditions and more image sequences of dynamic conditions, the paradigm was more authentic in terms of real-life emotional processing. To counteract the balance of the amount of image sequence between static and dynamic conditions, we selected 6 sequences in dynamic conditions that were next to the static conditions. These were distributed across the experiment.

Immediately after each sequence, 12 trials of the flanker task^47^ each 1200 ms (stimulus 200 ms and response window: 900, 1000 or 1100 ms) were presented to assess EF performance^48^. In the flanker task, a visual array of flag stimuli with the middle flag randomly surrounded by flags in the same direction (congruent) or in the opposite direction (incongruent). Participants responded to the direction of the middle flag as quickly and accurately as possible.

### EEG recording and processing

EEG was recorded from a 128- channel BioSemi EEG device with digital sampling rate at 512 Hz. All EEG channels were referenced offline to the algebraic average of left and right mastoids and notch filtered (55~65 Hz) to remove power-line noise. EEG signals were bandpass filtered between 1~40 Hz, using a two-way least squares finite impulse response filter in EEGLab^49^. Bad channels were identified and spherically interpolated. Then the data were epoched from 4 seconds before and 35 seconds (total image sequence and flanker task duration) after the sequence onset. Each epoch was visually inspected. Those epochs with obvious abnormal signal segments, such as head movement, were excluded for Independent Component Analysis (ICA). Then the extended Infomax ICA^50^, which was implemented in EEGlab as the default ICA algorithm, was utilized to detect and remove artifact contaminated by eye movements, muscle, and cardiac artifacts. We determined the ICA components by the ICA maps and also the power spectrum of the ICA component^49^. After removing the artifact components, the ICA source signals were transferred back to the original signal space, which was then used for the subsequent analysis.

The first half second of affective image viewing (0 to 500 ms) was removed from analysis to eliminate sensory transition effect. The average number of artifact-free seconds per participant was *M* = 79.90 (*SD* = 4.61), *M* = 79.80 (*SD* = 4.39), *M* = 238.40 (*SD* = 11.80) and *M* = 239.20 (*SD* = 9.20) for conditions of static-neutral, static-negative, dynamic-increase and dynamic-decrease respectively. There was no significant difference in the time length between static conditions (*p* = 0.910), and between dynamic conditions (*p* = 0.720).

**Figure 5:**
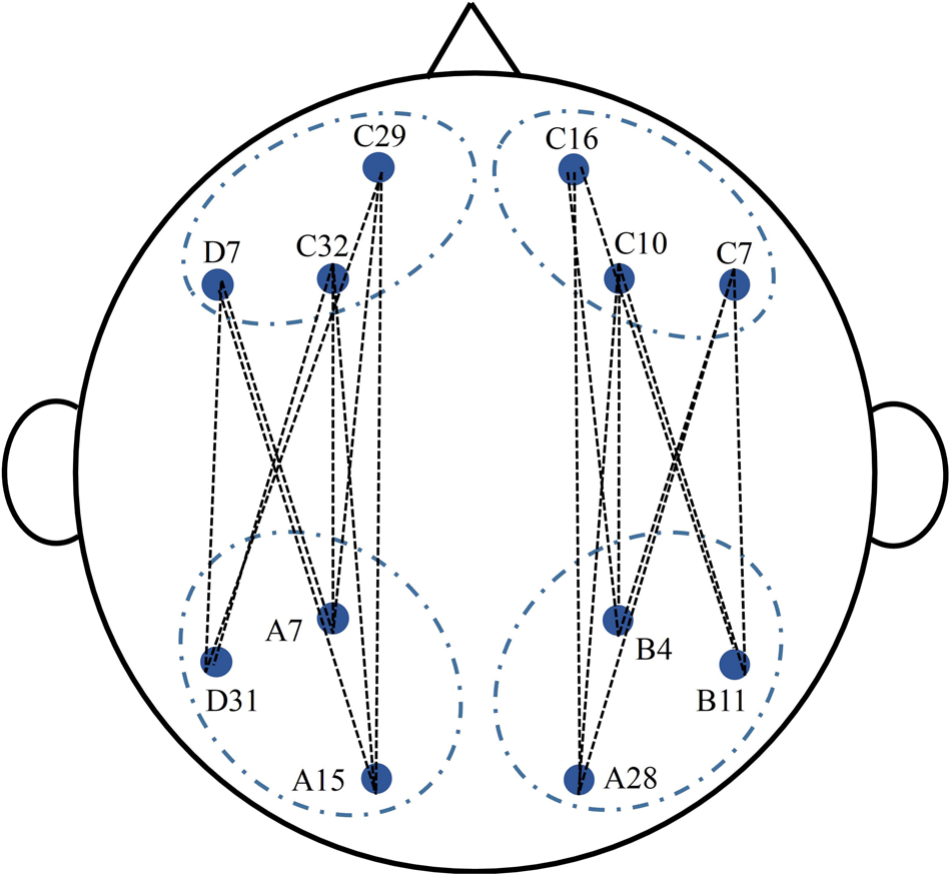
The electrodes used in the present study. Four clusters of electrodes were selected, with right frontal C16, C10, C7; left frontal C29, C32, D7; right parietal B4, B11, A28; left parietal A7, D31, A15. The dashed lines between electrodes represent functional connectivity.

Coherence measures the degree of covariance between two spatially distinct signals in prefrontal and posterior regions. We applied the magnitude-squared coherence, which was calculated by the cross-spectrum divided by the product of the auto-spectrum of the two signals. This measure includes information on the amplitude and phase. Electrodes distance is a factor that influences the effect of volume conduction. The spatial resolution of EEG is approximately 5 cm^51^, and the optimal distance between electrodes must be around 10–20 cm in human EEG-recordings to minimize the effect of volume conduction^52,53^. We confined our analyses to electrode pairs located no less than ~18 cm from each other to minimize the effect of volume condition. Following Miskovic and Schmidt (2010), four clusters of electrodes were selected, with right frontal C16, C10, C7; left frontal C29, C32, D7; right parietal B4, B11, A28; left parietal A7, D31, A15. Coherence scores of nine electrode pairs each were averaged to summarize interactions within the different brain regions respectively as shown in Fig. 5 (right hemisphere: C16-B4, C16-B11, C16-A28, C10-B4, C10-B11, C10-A28, C7-B4, C7-B11, C7-A28; left hemisphere: C29-A7, C29-D31, C29-A15, C32-A7, C32-D31, C32-A15, D7-A7, D7-D31, D7-A15). We calculated coherence throughout the image sequence (18000 ms) and then averaged all sequences for each condition.

Following the methods described in Miskovic and Schmidt^18^, artifact-free EEG data were submitted to a discrete Fourier transform with a Hamming window of 2000 ms width and 50% overlap, and Welch’s method was used to estimate the auto spectrum of itself and cross-spectrum between two signals. The cross-spectral coherence between two channels was calculated using the following formula in (1). (Note: *S*_*xy*_ denotes the cross-spectrum, *S*_*x*_ and *S*_*y*_ denotes the auto-spectrum; The E denotes the expectation across the repeated sequences). The coherence in beta frequency band ([14 30] Hz) and beta 1 frequency band ([14 20] Hz) were calculated throughout the image sequence (18000 ms) and then averaged all sequences for each condition.

1 $${R}_{xy}^{2}(f)=\frac{E{[{S}_{xy}(f)]}^{2}}{E[{S}_{x}(f)]\times E[{S}_{y}(f)]}$$

### Statistical analysis

We applied linear mixed effect models so that each individual was modeled as a random effect. We first used four experimental conditions to predict EEG coherence, and then reran the model with grouped static vs. dynamic conditions. Separate models were applied for each hemisphere. Since previous research revealed right hemisphere dominance in coherence changes during emotional processing, a priori, planned comparison was conducted on static-neutral vs. static-negative in the right hemisphere to test if our paradigm could replicate previous findings^18^.

Before we explored the image sequence condition effect on subsequent EF and the mediation role of EEG coherence, we predicted flanker reaction times (RTs) by image sequence conditions. Then RTs were predicted by coherence. Chronic stress was added as an interaction term with image sequence conditions. High and low chronic stress are plotted ±1 *SD* from the mean for descriptive purposes only in the Figures. Inferential analyses maintained the continuous nature of the chronic stress variable.

Flanker performance was calculated as RTs on incongruent trials, with RTs on congruent trials as a covariate. Trials with inaccurate responses or outlier RTs were deleted (3.14% of trials). We found that the first half of flanker trials (6 trials) followed by each image sequence had a stronger interaction effect with chronic stress while the effect decayed at the end given temporal separation from the emotional stimuli. Thus, we used the initial six flanker trials following the emotional stimuli in the following analysis.

## Supplementary information

Picture identification numbers from the International Affective Picture System (IAPS) used in this study

## Data Availability

The datasets generated during and/or analysed during the current study are available from the corresponding author on reasonable request.
