# Supplementary material for: Prefrontal-posterior coupling mediates transitions between emotional states and influences executive functioning
Source: Sci Rep. 2019 Jun 4;9:8252. doi: 10.1038/s41598-019-44624-2 (PMC6547671; doi:10.1038/s41598-019-44624-2)
Supplement: Supplementary file 1 — Picture identification numbers from the International Affective Picture System (IAPS) used in this study [file 41598_2019_44624_MOESM1_ESM.pdf]

Prefrontal-posterior coupling mediates transitions between emotional states and influences executive functioning

Yu Hao [1*], Lin Yao [2], Derek. M. Smith [3], Edward Sorel [4], Adam K. Anderson [5], Eric H. Schumacher [3], and Gary. W. Evans [1, 5]
[1] Department of Design and Environmental Analysis, Cornell University, Ithaca, NY, USA;
[2] School of Electrical and Computer Engineering, Cornell University, Ithaca, NY, USA;
[3] School of Psychology, Georgia Institute of Technology, Atlanta, GA, USA;
[4] Psychology Resource Group, Ithaca, NY, USA;
[5] Department of Human Development, Cornell University, Ithaca, NY, USA.
* Corresponding. yh597@cornell.edu

Appendix

Picture identification numbers from the International Affective Picture System (IAPS) used in this study

Neutral Images:

2026	2030	2038	2102	2235	2305	2381	2383	2384	2393	2396	2397	2411
2506	2512	2513	2570	2580	2593	2745.1	2840	2850	2890	4250	4605	5390
5471	5500	5510	5520	5530	5533	5534	5731	5740	6150	7000	7001	7002
7003	7004	7006	7009	7010	7012	7014	7017	7019	7020	7025	7026	7030
7031	7034	7035	7036	7038	7040	7041	7043	7045	7050	7052	7053	7055
7056	7057	7059	7080	7090	7100	7110	7130	7150	7160	7161	7170	7175
7179	7185	7187	7205	7217	7235	7300	7490	7491	7500	7513	7547	7705
7710	7950	8311	8312	9700								

Negative Images:

1080	1113	1205	1300	1301	1304	1310	1390	2115	2130	2276	2301	2456
2457	2691	2692	2694	2700	2750	2799	2810	2900	3019	3160	3181	3185
3220	3250	3300	5940	6190	6200	6200	6210	6213	6231	6260	6263	6312
6314	6561	6570	6825	6831	6834	6836	6838	7135	7137	8230	8485	9046
9080	9120	9145	9186	9250	9270	9291	9295	9321	9340	9342	9373	9395
9400	9403	9411	9425	9426	9427	9429	9470	9471	9495	9500	9530	9584
9590	9599	9610	9620	9621	9622	9623	9630	9832	9900	9901	9904	9909
9920	9926	9927	9930	9941								
